# Religion as Meaning-Making Resource in Understanding Suicidal Behavior in Ghana and Uganda

**DOI:** 10.3389/fpsyg.2021.549404

**Published:** 2021-06-07

**Authors:** Birthe Loa Knizek, Johnny Andoh-Arthur, Joseph Osafo, James Mugisha, Eugene Kinyanda, Charity Akotia, Heidi Hjelmeland

**Affiliations:** ^1^Department of Mental Health, Norwegian University of Science and Technology (NTNU), Trondheim, Norway; ^2^Department of Psychology, University of Ghana, Legon, Ghana; ^3^Department of Sociology and Social Administration, Kyambogo University, Kampala, Uganda; ^4^MRCV/Entebbe, Entebbe, Uganda

**Keywords:** suicidal behavior, meaning-making activity, Africa, culture, religion

## Abstract

Suicidal behavior is condemned by religions and tradition, and suicide attempts are criminalized by law in several African countries, including Ghana and Uganda. Suicide and suicide attempts may have severe consequences for both the entire family and the community. Religion is known to act as a protective coping force that helps people to make meaning and find comfort when dealing with stressful life events or situations like suicide. In this article, we focus on the cultural interpretations of the dominating religion in Ghana and Uganda, Christianity, and whether these affect attitudes toward suicidal behavior, meaning making, and coping possibilities for people who have attempted suicide or are bereaved by suicide. This article is based on data material from previous studies on the mentioned topics by the authors.

## Introduction

Suicide has been part of African cultures, and we find examples of traditional suicides in many ethnic groups over the continent (The Ethics of Suicide Digital Archive, 2015). On the other hand, historically one has assumed that African societies had low rates of suicide (Orley, 1970; Vaughan, 2013). Whether this is true or not in Ghana and Uganda is difficult to say as both countries do not have death registers, and no nationally representative studies on suicide have ever been undertaken, and WHO reports are figures generated by statistical modeling. Currently, attention is given to apparently rising suicide rates by both media and mental health professionals (Ovuga and Boardman, 2009; Quarshie et al., 2015), and Vaughan asks whether this is a symptom of moral panic or social crisis: “…in many of these reports on Africa suicide is represented as a symptom of a wider social and moral crisis, as a challenge to ‘traditional’ values, a sign of the ‘anomie’ consequent on modernization” (Vaughan, 2013, p. 233–234). Consequently, suicide is mainly discussed as a social or moral issue in the communities, as the meanings attributed to suicide are deeply intertwined with the normative discourses dominating society (Knizek et al., 2013). As this contextual meaning is of specific importance, it must be “…respected and understood, rather than referring to an imposed foreign model to explain the problem” (Patel et al., 1995, p. 219). During the last decades, more focus has been on research on suicidal behavior in some African countries and especially Ghana and Uganda. In this article, we focus on the findings from our previous studies on attitudes toward suicide and suicidal behavior among different groups (students, health professionals, lay people, police officers among others), as well as interviews with people who have attempted suicide or are bereaved by suicide in Ghana and Uganda, and try to understand them from an emic point of view in light of Park’s meaning-making model (Park, 2005, 2010). The reason for this focus is that it provides the most comprehensive qualitative material on this topic from the African continent.

Suicidal behavior is traditionally condemned, and attempted suicide is criminalized by law in several African countries, including Ghana and Uganda (Uganda, penal code act Cap. 120; Ghana, 1960 Criminal Code, Act 29, Section 57), which probably makes suicide the very last solution for individuals and, in addition, puts them and their relatives in an extremely vulnerable position as it attracts social stigma (Osafo et al., 2011). According to Adinkrah (2012), the person surviving a suicide attempt in Ghana is zealously prosecuted in criminal courts. The consequences of suicide and suicide attempts are manifold and affect the entire family and community (Mugisha et al., 2011; Osafo et al., 2011). Suicidal behavior often occurs when people find themselves in intolerable situations, where they cannot see any meaning in their lives and consequently lose hope (Mugisha et al., 2014). People may experience a crisis of meaning if fundamental relations or conditions are broken or when economic or ideological demands overstretch the capability of the individual (Mugisha et al., 2014). A person’s ability to make meaning determines whether the individual accepts or reorganizes his/her resources in order to cope and regain a feeling of control and security despite hardships they might experience (Park, 2010). Lately, researchers have focused on meaning making as a resource in health promotion (Ahmadi and Ahmadi, 2018).

A society’s normative ideological context forms the “basis” of the meaning construction that individuals adapt to their context and specific situations. Hall and Hill point out that “approaches to meaning making in suffering have been hampered by their failure to take into account the worldviews of the sufferers” (Hall and Hill, 2019, p. 469). Yet, studies have indicated that people’s idiosyncratic meaning systems are vital in daily life and may be of specific importance in understanding general patterns and individual differences in coping with adversity (Silberman, 2005). Thus, individuals’ idiosyncratic religious meaning systems function as a lens through which reality is perceived and interpreted, which “…influences formation of goals for self-regulation, affect emotions, and influence behavior” (Silberman, 2005, p. 645). As Africans are “notoriously religious,” as Mbiti (2006) puts it, religion has a heavy impact on meaning making generally and especially regarding suicide, which is regarded as a sin (Akotia et al., 2014). Consequently, individuals engaging in suicidal behavior as well as those close to them reflect along religious, normative lines, which traditionally condemn suicidal behavior (Kelleher et al., 1998). As religion is a communal meaning system, and suicidal behavior conflicts with this, suicidal individuals or those affected by suicide must reconsider their own situation and position toward God and society.

## Meaning Making and Religion

According to Frankl (2004), a sense of meaning is necessary to endure suffering. Others have taken up this line of thinking and developed it further (Park, 2005, 2010; Seligman, 2011; Proulx and Inzlicht, 2012; De Marinis, 2018). Most attention has been given to the model of Park (2005, 2010), which differentiates between global and situational meaning. The global meaning system is a personal worldview (Hall and Hill, 2019), which tends to be outside the immediate awareness of people, but has a powerful impact on their thoughts, actions, and feelings (Park, 2005). The global meaning system consists of beliefs, goals, and feelings and comprises views through which the individuals interpret their experiences (Janoff-Bulman, 1997; Mischel and Morf, 2003; Park, 2010). This meaning system is constructed through accumulated personal experiences and influences from the surrounding culture and their dominating belief systems (Baumeister, 1991). The broad views that are comprised within the global meaning system are, for example, justice, control, predictability, benevolence, personal vulnerability, and so on (Park, 2005, 2010). This global meaning system or worldview may be challenged by situational meanings, which are meanings “…in the context of a particular environmental encounter” (Park, 2010, p. 258) and come into action in the confrontation with potentially stressful events. In this situation, the individual must determine to what extent the situation is threatening, controllable, and which implications it may have for the future (Park, 2010). Comparisons with experiences from earlier situations or with other individuals in a similar situation might be used. During the process, the individual must decide whether the appraised meaning of the situation conflicts with the global meaning system or worldview. If a discrepancy between a situational meaning and the overall worldview or global meaning system is noticed, the individual has two options: either he/she must revise the global meaning system (accommodation) or the appraisal of the situation (assimilation). The larger the discrepancy between the global meaning system and the appraised situational meaning, the higher level of distress the person will experience: the world can seem incomprehensible and, hence, unpredictable, accompanied by a feeling of loss of control and meaninglessness. Meaning making is the process of restoring the global meaning system when it has been disrupted or violated (Park, 2005), which is in line with the coping model of Lazarus and Folkman (1984). As religion is highly influential in Africa, the global meaning systems of people will mostly be in accordance with their religious belief.

Religions are comprehensive meaning systems (Hood et al., 2005; Silberman, 2005; Newton and MacIntosh, 2013), which offer meaning to a variety of situations: “With respect to comprehensiveness, religions offer meaning to the entire scope of history, as well as to the full range of the lifespan and every aspect of human life. With respect to quality, religions provide answers to life’s deepest questions, offering hope and significance even in very difficult circumstances and the possibility of self-transcendence” (Hall and Hill, 2019, p. 470). According to Paloutzian (2005), Park (2005), and Silberman (2005), the meaning system integrates cognitive, affective, motivational, and behavioral elements in a psychological construct or processes, which cannot be understood independently of some element of faith (Paloutzian, 2005). Meaning making is an important process in which religion might contribute to coping in the context of encountering difficult life events (Hall et al., 2018). It is, however, not possible to see how religions work without looking at the content with an emic approach. What kind of religion influences people on which topics or in which situations? Below, we will focus on how Ugandan’s and Ghanaians’ confronted with suicide make meaning under the influence of religion.

### Religion and Attitudes Toward Suicide and Suicidal People

The religious meaning system heavily impacts attitudes toward suicide and suicidal people in Ghana and Uganda. In Uganda, approximately 84% are Christians, whereas about 14% are Muslim, and the rest are oriented toward traditional African beliefs (UBOS, 2014). In Ghana, there are 71.2% Christians (28.3% Pentecostal/Charismatic, 18.4% Protestant, 13.1% Catholic, and 11.4% others), 17.6% Muslims, 5.2% traditional beliefs, 0.8% others, and 5.2% none (2010 est.; Ghana Demographics Profile, 2019). Thus, we focus especially on Christians. In addition, in both countries, the normative ideological context is dominated by the fact that suicide attempts are criminalized by law, and the traditional communal value system regards suicide as a taboo (Gyekye, 1995; Mugisha et al., 2011; Knizek et al., 2013; Hjelmeland et al., 2014; Osafo, 2016). Attitudes consequently are influenced by three dominating moral systems, which in personal combinations form the attitudes toward a phenomenon that is highly problematic in all these value systems. In the legal system, suicide attempt is a crime; in religion, suicide is a sin, while it is considered an abominable act and a taboo in the traditional communal view (Knizek et al., 2013; Osafo, 2016). The three value systems are difficult to separate as they influence each other in the attitude formation.

Furthermore, it is important to clarify what religion is in this context. Even though the majority in both countries is Christian, one must consider the fact that Christianity might not be the same in Africa as it is in “the West”: “In Africa, religion does not represent a philosophy of life that searches for ultimate meaning, as it does for many Western Christians today. Rather, it represents a view of life that acknowledges the existence of an invisible world, believed to be inhabited by spiritual forces that are deemed to have effective powers over one’s life. The spirit world is considered to be distinct but not separate from the visible world of human beings. One implication of such a worldview is that the invisible world is an extension of the visible one, and people’s social relations extend into it. Therefore, it is important for human beings to maintain a good relationship with this spirit world” (Ter Haar, 2009, p. 28).

The connection with the spiritual world and the communal basis of the society leads to some caution about how people express themselves as it seems important to be conform to dominating meaning systems like religion and communal norms. Consequently, the personal views on suicide are difficult to unveil. The internal struggle between personal and communal views was described by Mugisha et al. (2014), as the participants openly displayed a dialogue between what they themselves thought and what the community attitude was. The dialogues almost always ended up with the participant expressing an attitude conforming with the community norms. There were a few exceptions in urban settings, where a personal attitude eventually was expressed (Mugisha et al., 2014).

Another important aspect in African religion is the expectation of reciprocity between the believer and the deity (Geyer and Baumeister, 2005; Mugisha et al., 2013; Akotia et al., 2014); people expect to be rewarded for good behavior and punished when they have repudiated doctrines. According to Wiredu (1996), the relationship between a god and his believers is utilitarian as their respect toward God is dependent upon whether or not they feel that He helps them to succeed (Akotia et al., 2014). Gyekye (1995) even described how a deity can lose his power and right to command his believers, if He does not fulfill his obligations toward his believers. African Christianity consequently can be understood as a form of contract, where both parts must fulfill their duties. The religious global meaning system, which is an immense force, therefore, has somewhat different components than Western Christianity.

In addition, religion as a dominating meaning system (Park, 2005) could be considered to have more power in this interdependent context than what is common in more independent settings. This was found, for example, in a study by Osafo et al. (2018), where they studied the attitudes of nurses and physicians toward suicide, and religion was the interpretive grid within which they expressed themselves.

The strong influence of religion on the attitudes toward suicide was found both in Uganda (Mugisha et al., 2013) and Ghana (Osafo et al., 2011, 2018). Osafo (2012) interviewed various groups as, for example, psychology students, psychologists, nurses, and lay persons and found that suicidal behavior mainly was perceived as a moral issue with moral meanings. In one study, 27 lay persons from both rural and urban settings were interviewed (Osafo et al., 2011). All participants considered themselves religious and expressed a religious identity (24 Christian and three Muslim). Almost all the Christians cited the Bible when explaining their attitude or explained it in more general terms, for instance: “My training in the Lord was to the extent that I saw suicide as something that is not good” (Osafo et al., 2011, p. 493) or “the Bible does not permit you to go and pick a rope and hang yourself! And so we must respect that” (Osafo et al., 2011, p. 495). While the first participant expressed a personal attitude through the personal pronoun “I,” which, however, conformed with the Christian doctrine, the second participant immediately showed his unity with the general meaning system by choosing the inclusive pronoun “we.” The general reasoning was that because God gave life, he also is the only one who can take it. Life is sacred and must be preserved under all conditions, as expressed by one participant: “Life is much more precious than what one might be going through and thus no matter what, one must not destroy it” (Osafo et al., 2011, p. 495).

The same categorical attitudes toward suicide were seen in Uganda. Mugisha et al. (2013) conducted a study with 28 focus group discussions with lay people and 30 key informant interviews with political and traditional healers on their attitudes, as Baganda, toward suicide and suicidal people. Life was perceived as God given and consequently sacred. Nobody should consider suicide an option even when suffering atrocities, for instance, as expressed by a participant in a focus group discussion: “The Bible and the Koran are clear on this; you are not allowed to kill; including oneself (Mugisha et al., 2013, p. 352).

The global religious meaning system in Ghana and Uganda seemed clear about the unacceptability of suicide. However, in Osafo et al. (2011) study, religion’s inherent value system also was a motivator for helping people in suicidal crisis, despite the principal rejection of suicide as an option in a difficult situation. The remedy they mainly used in order to (re)install meaning and hope for the suicidal person was the script and/or prayer as expressed by this rural woman:

“A woman came and told me she has crisis in her marriage. I told her ‘keep the words of your Bible and pray in the night’. She came to me and thanked me the next morning for my advice to her last night” (Osafo et al., 2011, p. 496).

Here it is again necessary to remember that religion as a source for the global meaning systems in these countries is different than what is usual in Western settings, where it is a philosophy of life. In Ghana and Uganda, God is much more present, and there is an immense trust in his power and the ability to reach him directly through prayer: “They believe in the power of prayer, which gives them unmediated access to a God who listens to them and answers their requests, a God who communicates directly with believers, perpetuating a tradition of direct communication with the spirit world” (Ter Haar, 2009, p. 51).

In this intimate relationship with and trust in God, consolation can be found as the meaning is left to God. It does not matter whether the individual is able to make meaning in the situation, as this might all be part of the divine plan that humans are unable to comprehend. The meaning consequently is with God. The downside of this is that one not only might expect support from God but also punishment for acts like suicide, which are perceived as against His will (Mugisha et al., 2013). Consequently, the prescribed ways of living include avoiding provocation of God.

The religious meaning system builds the basis of the attitudes toward suicide and suicidal people. The global and situational meanings seem to be in harmony, maybe even identical, in general, if one accepts unconditionally to be part of God’s plan and acts accordingly. The problem, however, comes into existence when a person acts against the will of God and, consequently, the public opinion. In this highly religious prescriptive context, people who attempt suicide or are bereaved by suicide struggle to find meaning with the acts and their social position.

## Attempted Suicide and Meaning Making

In Ghana, some studies have focused on suicide attempts (Akotia et al., 2014, 2019; Osafo et al., 2015; Asare-Doku et al., 2019). The question of meaning making in the situation by means of religion came to the fore in the study by Akotia et al. (2014) where 30 people having attempted suicide were interviewed while in the hospital. Twelve men and 18 women between 18 and 46 years participated in the study. With the exception of two who were Muslims, all were Christians, and they generally ascribed to the dominating meaning system, which reserves the possibility to take life a prerogative for God, even though they have offended this doctrine: “I know I do not own my life … it is God who gives life and He should be the only one to take it … you see it is not good” (Akotia et al., 2014, p. 439). This man has broken one of the most important rules of the dominating religious meaning system and has a dissonance between attitudes and behavior (cognitive dissonance, Festinger, 1957). According to Park (2005, 2010), being in a situation like this requires either a re-appraisal of the actual situation (assimilation) or a reconsideration of the global meaning system (accommodation). Both processes were seen in the study of Akotia et al. (2014), with assimilation being the most frequently employed.

During the assimilation process, participants questioned not the global meaning system, but mainly whether they had fulfilled their obligations to God and whether their situation might be a result of this. The basis of this interpretation is the idea of a reciprocal relationship between God and his believers (Mugisha et al., 2013). If the believers lead their life according to God’s will, they expect to get a good life. In accordance with this, we must understand the following reasoning:

“I told you that I used to go to church, but I stopped. If I were still in church and this occurred, I would say that trials are common to humans but because I stopped going to church, look at what happened to me! So, I will say that not going to church also caused the attempted act of suicide” (Akotia et al., 2014, p. 441).

The religious meaning system still was recognized as valid, and the participant found the problem being his neglect of the obligations toward God. This way of thinking was common among most of the participants, but not all. A few participants changed their view on religion (accommodation) and did not accept their situation as part of God’s divine plan. These participants blamed God for their situation and changed their global meaning system; from being good, God was now experienced as deceitful. They felt that they had done everything to please God, but they did not get their expected reward but misery instead. These participants felt betrayed:

“…I bought the drug because I was thinking that for me who has given my life to God, some things should not be happening in my life, and every day … I wanted to end it all. All the things happening to me, I believe God should have fought for me but that did not happen” (Akotia et al., 2014, p. 443).

The participants blamed God for either causing or allowing their suffering and consequently changed their global religious meaning system, which until then had implied a supreme and good God.

The feelings that followed the assimilation or accommodation process were different. While assimilation was followed by guilt and self-condemnation (Akotia et al., 2014; Osafo et al., 2015), accommodation led to feelings of being let down or betrayed and could result in anger and mistrust toward God (Exline and Rose, 2005), or to an entire abandonment of the religious meaning system (Altemeyer and Hunsberger, 1997). Whether a person who has attempted suicide chooses assimilation or accommodation might have huge consequences for their mental health. Those who engaged in assimilation and saw their act as a sin and asked for forgiveness in order to restore harmony between the global and the situational meaning still had a source of hope for the future. Consistent with views of worse outcomes in an accommodation process in which the worldview is altered to a nihilistic vision of the world and one’s place in it (Hall and Hill, 2019), persons who went for accommodation ended up embittered and without hope (Akotia et al., 2014), and lost their religious faith as a coping mechanism (Osafo et al., 2015). Such an outcome could be suicidogenic given the link between hopelessness and suicide (Shneidman, 1993). The implication is that besides religion being a powerful meaning system, which can give people direction and comfort, it can also have serious consequences in the form of further struggles and distress (Exline and Rose, 2005; Akotia et al., 2014). In addition to their internal struggles, those who had attempted suicide were stigmatized by the family and/or community, and the loss of social support aggravated the loss of faith since nothing was left for those who had employed accommodation. When accommodation leads to abandonment of faith or an assumed deceitful God who undermines the feeling of predictability, it is difficult to accept it as a coping mechanism; it is rather the opposite. The concept of accommodation, thus, seems problematic. The majority who had used assimilation had preserved a powerful coping mechanism as well as a possible reestablishment of the social support.

As suicidal behavior not only affects the individual who intentionally has chosen this action but also the people close to him/her, it is interesting to look at how people bereaved by suicide handle the situation they involuntarily found themselves in.

## Meaning Making After a Suicide

To date, only one study in Ghana (Andoh-Arthur et al., 2017, 2018, 2019) and one in (post conflict northern) Uganda (Kizza et al., 2012) have focused on people bereaved by suicide. None of these studies looked specifically at the meaning making or coping mechanisms of the bereaved, but it is possible to get some insight by means of the participants’ reactions to the suicides in the study in Ghana. The religious meaning system and the public opinion have, as mentioned, an immense power in Ghana and is culturally adapted, so a nephew of a man who had taken his life could choose to be faithful to his religion and blame the deceased person:

“You see, we are sent here for a purpose, right? That is what the Bible teaches me as a Christian. I do not know what the other religions teach, but I was sent on this earth for a purpose. If I finish my job, I will be called back; so I do not think this [suicide] is an act that should not be condemned. It means you are a coward; you did not finish the race” (Andoh-Arthur et al., 2019, p. 3).

This bereaved nephew distances himself from his deceased uncle and describes him in an offensive way in an assimilation process. In a way, he has suffered a double loss: the actual death and a character killing. The latter, however, preserves his religious faith and probably secures his social position. Assimilation was prominent also after a suicide but had huge familial consequences, as earlier valued family members were abandoned through this meaning-making process, and consequently, intra familial bonds had to be reconstructed. However, some of the bereaved were able to employ a cultural interpretation of the global religious meaning system and avoided a discrepancy between a global meaning system and a situational meaning:

“I go to church, and I believe in spiritual things, so I think it was spiritual. No one would get up to go and kill him or herself. I think it is spiritual, so they [family of deceased person] had to find out, but they did not” (Andoh-Arthur et al., 2019, p. 4).

This friend emphasizes that he adheres to his religious duties and believes in a spiritual world, which he claims as reason for his friend’s death. The friend consequently is innocent, while spiritual forces, which are part of his global meaning system, must be blamed. Arguing this way both keeps the dominating meaning system intact and preserves the positive memory of the friend. This case is interesting as it shows exactly what Hall and Hill (2019) have claimed: it is necessary to look closer into the content, when studying how meaning making happens by means of religion. The friend in this case is a Christian, but his understanding of Christianity seems to be rooted in the cultural belief of an invisible, spiritual world, which has had an evil influence on the deceased person. Christianity for him clearly means more than the Western Christians’ search for an ultimate meaning in life (Ter Haar, 2009) and can be understood as cultural meaning making in a religious frame.

## Culture Specific Religion As Meaning-Making

Suicide is the outcome of a crisis or psychache (psychological pain, Shneidman, 1993) and evokes powerful reactions of those affected. Forceful attitudes from the community form the normative context of suicidal behavior, which, in a mainly interdependent society, has a huge impact. “All cultures have canonical narratives of suicide, that is, taken-for-granted storylines that provide members of the culture with meaning systems for understanding suicides” (Marecek and Senadheera, 2012, p. 59). In contexts where suicide attempt is a criminal offense, traditional values deem suicide an abomination and a taboo, and religion, which has enormous power and influence, condemns suicidal behavior, people considering suicide might feel additional pressure. They endanger their entire family at the same time as they see no other solution. As religion is part of their lives, one must consider how suicidal people and those affected by suicide employ their religion during the entire process in order to make meaning out of something that shakes their entire universe.

Religion, as a meaning system, is deemed a “powerful source of meaning under even the most testing circumstances, such as when stressful events cannot be repaired through problem solving strategies” (Silberman, 2005, p. 647). According to Park (2005) and Shaw et al. (2005), people do better under crisis, and even better under high levels of stress, if they are religious (Pargament et al., 2006). Religious coping has been shown to be the strongest predictor of positive psychological change after traumatic events (Prati and Piantroni, 2009), while negative religious coping (Pargament et al., 2004) and spiritual struggles (Werdel et al., 2014) have negative outcomes. The question discussed in this article is whether this is true also if the crisis leads to acts that conflict with the religious doctrines and whether the cultural religious context allows a different latitude than what is shown in etic approaches. As outlined by Hall and Hill (2019), approaches to meaning-making have failed to consider the worldviews of the suffering, which means that specific cultural meaning-making possibilities might have been overlooked.

The global meaning or worldview (Hall and Hill, 2019) of people in Ghana and Uganda is influenced by religion as a comprehensive meaning system, which influences global beliefs about the world and the self, as well as life goals (Baumeister, 1991; Emmons, 1999). However, religion also influences situational meanings: “…including initial appraisal of events as a loss, threat, or challenge, as well as attributions regarding the reason why the event occurred. While global religious beliefs provide the framework for these attributions, religions also offer specific beliefs about suffering that may be part of situational meaning” (Hall et al., 2018, p. 78). If one, for example, believes that suffering on earth is part of God’s plan and that it promises a good afterlife, suffering might be experienced totally different than believing in no comfort in the afterlife or no afterlife at all. It is important also to consider the differences in religion as studies have demonstrated that the relationship between religious coping is moderated by religious affiliation (Goodman et al., 1991; Alferi et al., 1999; Osborne and Vandenberg, 2003; Williams et al., 2006; Wojtkowiack et al., 2010; Hall et al., 2018). According to Hall et al. (2018), this results in different religions’ specific reinforcement of certain coping strategies, which may shape anticipated outcomes of those coping methods. From our work, we might, in keeping with Ter Haar (2009), go even further and claim that the religions, in addition, are culturally adapted. In Ghana and Uganda, Christianity is the most prominent religion. Christianity has, in this context, developed specific features with the reciprocal, utilitarian relationship with deity and the direct access to God and the spiritual world. This culture-specific understanding of Christianity seems to pave the way for specific religious meaning-making coping. While most people condemned suicide and adhered to the religious doctrines, we also saw global meaning systems, which did not create discrepancies with the actual situation. Though Hall et al. (2018) point at with-in group differences in religious practices, we must consider the possibility that these differences might be grounded in completely different ontologies. The newer religions as Christianity or Islam still reflect “…elements of strong continuity with the continent’s original religious traditions. This pattern of understanding is a social fact that should impose itself on any coherent analysis. Hence, (…), it becomes important to render conventional Western ideas concerning religion in Africa in a symbolic language that takes African realities into account, rather than simply applying the vocabulary and concepts that conventionally underlie Western secular thought” (Ter Haar, 2009, p. 99).

We claim that an etic approach to religious meaning making in crisis, applying Western models of religious meaning-making coping, has not enough explanatory potential to capture all aspects of the meaning-making coping in the Ghanaian and Ugandan context. Most of those who had attempted suicide and those bereaved by suicide had employed what looked like assimilation and maintained their faith and position in the community. Fewer people used accommodation and lost their coping resource. This is in line with what has been found earlier (Janoff-Bulman, 1992). Others have argued that accommodation might be more common when the stressor is major and irreversible (Brandtstädter, 2002). We saw, however, examples of something totally different, where discrepancies did not arise, as the content of the global meaning system gave room to both keeping it intact and simultaneously to retain warm feelings and a good memory of the deceased person. This is in line with Pargament (2011), who emphasizes that a non-reductionist view considers that “… either specific religious beliefs (e.g., belief in an afterlife) influence the relationship or the connection of religious coping on well-being is unique to religious practices” (Hall et al., 2018, p. 82). We therefore must consider both etic and emic factors in coping processes for a better understanding. In 2002, Pargament called for a new type of research, which would be able to answer the questions of the effects of “…particular types of religion, for particular situations, within particular social contexts and according to particular criteria of helpfulness” (Pargament, 2002, p. 178). In our opinion, much more research on the specific meaning systems in their cultural context is needed, before it is possible to understand peoples’ efforts to cope with suicide and suicide attempts.

## Concluding Remarks

Suicide is a devastating event that shatters the life of many people, and it is necessary to understand how people make meaning when they are confronted with suicide or suicide attempts in order to establish effective support. Meaning making contemporarily attracts increasing attention in order to understand how people cope in difficult situations (Paloutzian and Park, 2005; Ahmadi and Ahmadi, 2018). In Ghana and Uganda, religion is a powerful force in the life of people, which permeates their meaning-making processes. In Ghana, promising collaboration between psychologists and religious bodies are ongoing, among others, through the Center for Suicide and Violence Research (CSVR),^1^ which is a registered research center run by academics at the Department of Psychology, University of Ghana. Their special interest is to prevent suicide/self-harm behaviors among young people and also reduce violence of all forms against people. They work with knowledge generation (through research) to inform training, advocacy, and policy direction. The center builds collaborative relationships with various civil society groups, government institutions, and international agencies with similar focus on preventing suicide/self-harm and end violence. The collaboration with religious bodies is included in this work. The religious force in people seems to be a valuable resource to employ, and the Ghanaian example is worthy of imitation and further development. However, reductionist Western meaning models seem not to capture religions’ cultural complexity entirely, and new emic approaches are called for in order to uncover the full range of valuable support resources.

## Author Contributions

The first author (BK) collected earlier studies of the group under the meaning-making frame and wrote up a first draft of the article. The other authors (JA-A, JO, JM, EK, CA, and HH) commented critically the first draft. The first author revised the draft upon their comments and resent it to the other authors, who once more commented detailed. Based on these comments, the final version of the article was drafted. All authors contributed to the article and approved the submitted version.

### Conflict of Interest

The authors declare that the research was conducted in the absence of any commercial or financial relationships that could be construed as a potential conflict of interest.

## Footnotes

^1^https://www.end-violence.org/members/centre-suicide-and-violence-research-csvr-university-ghana
